# Diagnostic Accuracy and Real-Life Advantages of the MONA.health Artificial Intelligence Software in Screening for Diabetic Retinopathy and Maculopathy

**DOI:** 10.3390/diagnostics16050730

**Published:** 2026-03-01

**Authors:** Martina Tomić, Romano Vrabec, Toma Babić, Kristina Kljajić, Tomislav Bulum

**Affiliations:** 1 Department of Diabetic Eye Complications, Vuk Vrhovac University Clinic for Diabetes, Endocrinology and Metabolic Diseases, Merkur University Hospital, 10000 Zagreb, Croatia; martina.tomic@kb-merkur.hr (M.T.); romanovrabec@yahoo.com (R.V.);; 2 Department of Diabetes and Endocrinology, Vuk Vrhovac University Clinic for Diabetes, Endocrinology and Metabolic Diseases, Merkur University Hospital, 10000 Zagreb, Croatia; 3 School of Medicine, University of Zagreb, 10000 Zagreb, Croatia

**Keywords:** diabetic retinopathy and maculopathy screening, artificial intelligence, standard fundus photography, diagnostic accuracy, real-life advantages

## Abstract

**Background**/**Objectives:** We aimed to evaluate the diagnostic accuracy of the MONA.health artificial intelligence (AI) software (Version 1.0.0; MONA.health, Leuven, Belgium) and compare its advantages in screening for diabetic retinopathy (DR) and diabetic macular edema (DME) with standard fundus photography. **Methods:** This cross-sectional, real-life instrument validation study was conducted at the Vuk Vrhovac University Clinic in Zagreb during routine DR screening and included 296 patients (592 eyes) with diabetes. Following standard fundus photography using a 45° Zeiss VISUCAM NM/FA camera (Carl Zeiss Meditec AG, Jena, Germany), each patient also underwent imaging with an automated portable retinal camera (NFC-600, Crystalvue Ophthalmic Instruments, Taoyuan City, Taiwan). Two retina specialists independently graded images from the standard camera, while images from the NFC-600 were analyzed using the MONA.health AI software. **Results:** Among the 592 eyes, human grading identified 81 with any DR, including 17 with mild NPDR, 64 with referable DR (moderate/severe NPDR or PDR), and 13 with DME. The MONA.health AI software identified 65 eyes with referable DR and 19 with DME. For MONA DR screening compared to the standard fundus camera, the area under the curve, sensitivity, specificity, positive predictive value, negative predictive value, positive likelihood ratio, negative likelihood ratio, kappa agreement, diagnostic odds ratio, and diagnostic effectiveness were 99.74%, 100%, 99.81%, 99.33%, 100%, 528.00, 0.00, 0.99, infinity, and 99.85%, respectively. For MONA DME screening, these metrics were 97.97%, 100%, 98.95%, 85.93%, 100%, 95.67, 0.00, 0.81, infinity, and 99.02%, respectively. The MONA AI screening process required 1 day of training and approximately 5 min for image capture and analysis, compared to 7 days of training and 13 min for image acquisition and grading with the standard method. **Conclusions:** These findings demonstrate that the MONA.health AI software matches the accuracy of standard fundus photography for screening and early detection of referable DR and DME, while offering a faster, simpler, and more user-friendly workflow that significantly reduces the time to obtain screening results.

## 1. Introduction

More than 40% of individuals with diabetes develop diabetic retinopathy (DR), which remains the leading cause of preventable blindness among the adult working population [1]. The global prevalence of diabetes is increasing rapidly, with projections indicating that the number of affected individuals will reach 783 million by 2045. Consequently, the prevalence of DR is also rising worldwide, particularly in developing regions, with cases expected to increase from over 100 million currently to more than 160 million by 2045 [2]. Among those with DR, over 10% present with advanced forms, such as proliferative DR (PDR) and diabetic macular edema (DME) [3]. These statistics underscore the importance of early identification of DR during its asymptomatic phase, as prompt initiation of appropriate treatment can substantially reduce the risk of visual impairment and blindness.

Population-based screening programs for DR are widely regarded as the most effective and economically sustainable strategy for preventing diabetes-related vision loss [4,5]. Standard DR screening protocols typically involve an assessment of visual acuity prior to pupil dilation, followed by a retinal evaluation sufficient for accurate DR staging [6]. However, manual interpretation of retinal images by ophthalmologists is not feasible for large-scale screening of the diabetic population. The widespread availability of affordable fundus imaging devices has facilitated alternative approaches to DR screening [7]. Trained nurses or certified photographers can capture retinal images with fundus cameras, producing high-quality photographs suitable for further analysis. These images may be evaluated by ophthalmologists or processed using fully automated artificial intelligence (AI)-driven diagnostic systems for DR detection and grading [8,9]. AI-based systems can serve as low-cost, point-of-care tools for DR detection and help alleviate the burden of diabetic eye screening [10]. Such systems provide immediate screening outcomes by determining whether a patient requires referral to a specialist, offering a cost-effective alternative to universal ophthalmologist-based screening. The first fully autonomous AI system for DR detection received approval from the United States Food and Drug Administration (FDA) in April 2018 [11]. Since then, numerous additional AI-based screening tools have been developed and integrated into clinical and screening workflows [12,13,14].

Many AI models are trained on datasets that lack sufficient diversity. Consequently, their predictive accuracy often fails to generalize across different populations, healthcare settings, or geographic regions. Performance evaluations within specific subgroups, such as those defined by ethnicity, are rarely conducted, raising concerns about the reliability of applying these models to previously unencountered patient populations [15]. As a result, the high performance reported in scientific studies is often not reproduced in real-world clinical settings, potentially reinforcing or even amplifying existing healthcare disparities and algorithmic biases. Certain limitations may be addressed by conducting prospective clinical studies that incorporate predefined metadata and ensure balanced representation of relevant subpopulations [16]. Nevertheless, such trials are resource-intensive and time-consuming, and are typically restricted to a small number of clinical sites, thereby limiting the scope of model validation. Automated retinal image analysis systems, including MONA.health, demonstrated high sensitivity for medium- and high-risk DR in a real-world screening service that included over 1 million images, with equitable performance across population subgroups by age, sex, and ethnicity, compared with the reference standard of human grading [17].

This study aimed to evaluate the diagnostic accuracy of the MONA.health AI software and to compare its practical advantages in screening for DR and DME with those of standard fundus photography within routine clinical practice.

## 2. Materials and Methods

### 2.1. Study Design and Ethics

This cross-sectional, real-life instrument validation study was implemented as a proof-of-concept (POC) project at the Vuk Vrhovac University Clinic for Diabetes, Endocrinology and Metabolic Diseases, Merkur University Hospital in Zagreb, Croatia. The study adhered to the Declaration of Helsinki and received approval from the Ethics Committee of Merkur University Hospital (protocol number 03/11-299, approval date: 20 February 2023). All participants were provided with written and oral information regarding the study and signed informed consent forms.

During the three-month POC project, from March 2023 to June 2023, and in accordance with the POC agreement, the Department of Diabetic Eye Complications received a Dell Latitude E5470 laptop (S/N 8P32RF) (Dell Inc., Round Rock, TX, USA) and an automated portable retinal camera, the NFC-600 (S/N A002-124-0020), from MONA.health (Leuven, Belgium). MONA.health did not participate in the analysis or interpretation of the project results to prevent any conflict of interest.

### 2.2. Patients

A total of 296 patients (592 eyes), of both genders and older than 18 years, with type 1 diabetes mellitus (T1DM) and type 2 diabetes mellitus (T2DM), referred to the Department of Diabetic Eye Complications for DR screening between March 2023 and June 2023, were randomly selected for inclusion in the study. At the inclusion visit, the primary investigator, M. T., obtained a brief medical history regarding diabetes and other ocular conditions. Patients with diseases affecting the posterior segment of the eye, such as macular degeneration or retinal vascular occlusions, were excluded. Additionally, patients with conditions that restricted fundus visualization and photography, including previous ocular trauma, acute infections, ocular surface diseases, mature cataracts, opacities, vitreous hemorrhages, or poor cooperation, were not included.

### 2.3. Fundus Photography and Diabetic Retinopathy Grading

After signing the informed consent and pupil dilation with 0.5% tropicamide eye drops, each patient first underwent standard fundus photography with a 45° Zeiss VISUCAM NM/FA camera (Carl Zeiss Meditec AG, Jena, Germany), followed by fundus photography with a retinal camera NFC-600.

**Figure 1:**
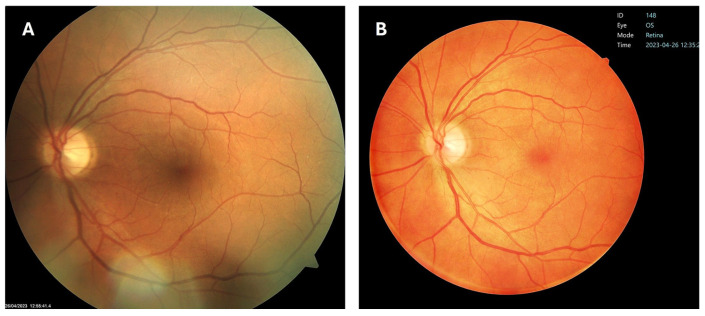
Images of the same patients taken by standard VISUCAM NM/FA camera (**A**) and NFC-600 retinal camera (**B**).

In our routine daily retinological DR screening program, using a standard 45° Zeiss VISUCAM NM/FA camera, we perform color fundus photography of two fields (macular and disc/nasal) per eye according to the EURODIAB retinal photography methodology [18]. Two-field photography has the advantage of detecting DR in the nasal retina that single-field photography could otherwise miss. For this study, however, only one image (macula-centered field) per eye was captured and analyzed with the standard 45° Zeiss VISUCAM NM/FA camera, covering the temporal area and optic disc, to match the images obtained with the portable, fully automatic, non-mydriatic retinal camera NFC-600 (Crystalvue Ophthalmic Instruments, Taoyuan City, Taiwan), which also provides a 45° field of view (Figure 1).

Images captured with the standard VISUCAM NM/FA camera were independently graded and assigned a DR level by two retina specialists (M. T. and R. V., or M. T. and T. Babić) using the Proposed international clinical DR and DME disease severity scales (ICDR classification) [19]. As both specialists assigned identical grades, a third grader was not required. Prior to grading, all images from the VISUCAM NM/FA camera were assessed as excellent or good quality, with none deemed low-quality or unreadable. This ensured comparability in image quality with those obtained from the fully automatic NFC-600 camera and minimized potential device-related confounding factors.

**Figure 2:**
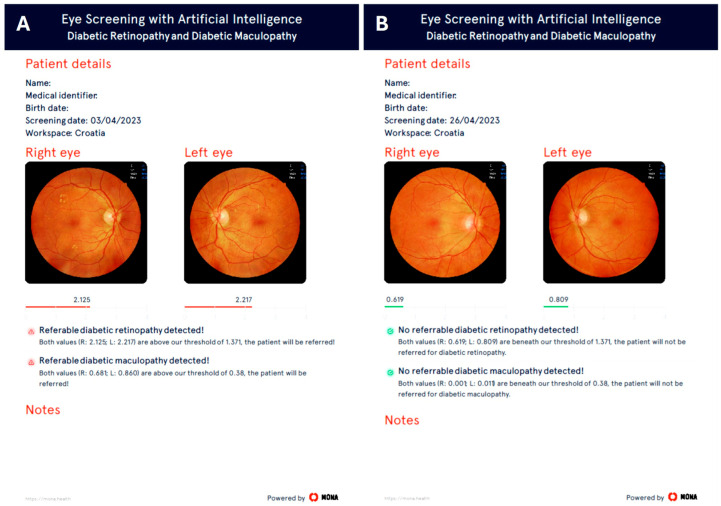
Eye screening with MONA.health artificial intelligence with threshold values for referable retinopathy and maculopathy (**A**) and no referable DR and DME (**B**).

In contrast, images from the NFC-600 camera were uploaded to the MONA Cloud, where they were analyzed and graded for DR and DME using the MONA.health artificial intelligence (AI) software. The MONA.health diabetic eye screening software, MONA DR DME (Version 1.0.0; MONA.health, Leuven, Belgium, https://mona.health), utilized in this proof-of-concept project, was previously described in detail by Peeters, F. et al. [20]. The software requires one fundus image per eye, centered between the macula and the optic disc, for algorithmic processing and generates three diabetic eye screening results per patient: DR, DME, and a combined outcome. The core of the MONA.health screening software comprises two deep learning ensembles, one for DR and one for DME. Each ensemble incorporates multiple convolutional neural network architectures (such as ResNet, EfficientNet, Xception, InceptionV3, DenseNet, and VGG) that differ in structure and training parameters; their outputs are averaged to produce a final prediction. The DR ensemble performs regression to estimate disease grade, while the DME ensemble predicts the probability of disease presence, with both models operating in parallel. MONA.health provides detailed performance metrics to validate the training and operation of its AI algorithm. Technical evaluations of the MONA software report indicators such as sensitivity, specificity, area under the receiver operating characteristic (ROC) curve (AUC), performance validation, and comprehensive reports. The platform also offers real-time error functions, such as loss curves, during training. These metrics enable users to assess model accuracy and evaluate training success. The software is designed to screen for referable DR and the presence of DME, thereby identifying patients who require referral to an eye specialist. Consistent with clinical practice, the AI screening software applies the ICDR classification for grading DR and DME severity [19], categorizing moderate non-proliferative DR (NPDR; grade 2), severe NPDR (grade 3), and proliferative DR (PDR; grade 4) as referable DR. DME (M1/M2) with hard exudates or thickening located within or closer than one disc diameter to the fovea is classified as referable maculopathy (DME). The AI screening software threshold values for referable DR and DME are set at 1.371 and 0.380, respectively, both indicated in red. Values below these thresholds indicate no DR (grade 0) or mild NPDR (grade 1), and the absence of DME (M0), indicated in green (Figure 2).

### 2.4. Statistical Analysis

Statistical analyses were conducted, and graphical representations were generated using Statistica™ 14.0.1.25 (TIBCO Inc., Palo Alto, CA, USA). The Kolmogorov–Smirnov test was applied to evaluate data normality, while the Levene test assessed variance homogeneity. Descriptive statistics were reported as median (min–max) for continuous variables and as counts (percentages) for categorical variables. Differences in continuous variables were analyzed using the Mann–Whitney test for two independent groups and the Wilcoxon test for two dependent groups. Nonparametric tests were selected because the homogeneity-of-variance assumption was violated. The Chi-square test was employed to compare categorical variables. Receiver operating characteristic (ROC) curves, area under the ROC curve (AUC), sensitivity, specificity, predictive values, likelihood ratios, kappa agreement, diagnostic odds ratio, and diagnostic effectiveness (accuracy) were calculated to evaluate the performance of the MONA.health artificial intelligence software in screening for diabetic retinopathy and maculopathy, and to determine its diagnostic accuracy in detecting referable DR and DME compared to standard fundus camera photography. Statistical significance was defined as a *p*-value less than 0.05.

## 3. Results

### 3.1. Demographic Data and Prevalence of Diabetic Retinopathy and Maculopathy

A total of 296 diabetic patients (592 eyes), comprising 160 males and 136 females with a median age of 63 years (range 19 to 81), were included in this cross-sectional, real-life instrument validation study. Their demographic data stratified by diabetes type are presented in Table 1. Patients with type 1 diabetes were significantly younger (*p* < 0.001) and had a longer disease duration compared to those with type 2 diabetes (*p* = 0.003). In contrast, no significant difference in gender distribution was observed between the diabetes type groups (*p* > 0.05).

Among the 592 eyes included in the study, human grading identified 81 eyes (13.7%) with any DR. Of these, 17 had mild NPDR and 64 had referable DR (moderate or severe NPDR or PDR), while 13 had DME.

The MONA.health AI software identified 65 eyes with referable DR and 19 eyes with DME. For eyes with referable DR, the median treasured value was 1.945 (range: 1.391 to 3.554), while in eyes without DR, the median treasured value was 0.237 (range: 0.021 to 0.972). For referable DME, the median treasured value was 0.805 (range: 0.399 to 0.954), compared to 0.001 (range: 0.000 to 0.326) in eyes without DME.

### 3.2. Diagnostic Accuracy of the MONA.health Artificial Intelligence in the Detection of Referable DR

**Figure 3:**
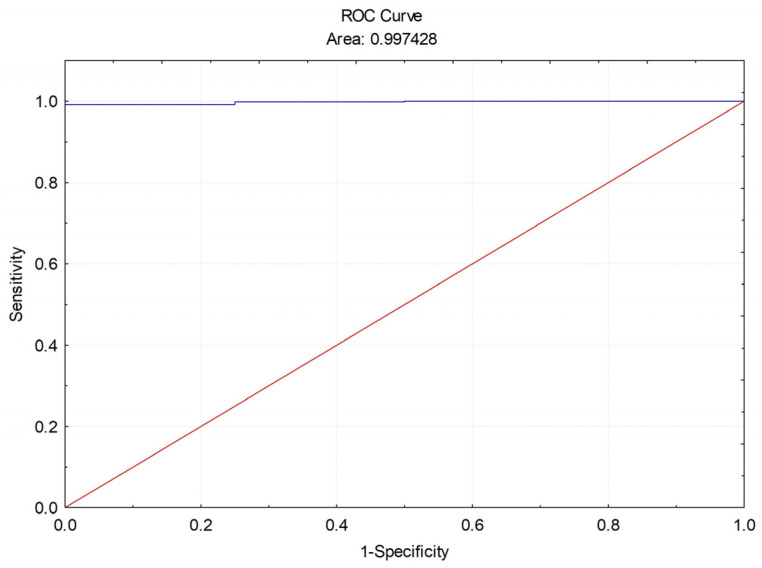
ROC curve of referable DR detection by the MONA AI vs. standard VISUCAM NM/FA Zeiss fundus camera.

The shape of the ROC curve and the AUC of 99.74% demonstrate the strong discriminative capability of the MONA.health artificial intelligence system for detecting referable DR, compared with the standard VISUCAM NM/FA Zeiss fundus camera and human DR grading (Figure 3).

Additional diagnostic accuracy metrics for referable DR detection by the MONA AI, in comparison with the standard VISUCAM NM/FA Zeiss fundus camera and human DR grading, are presented in Table 2.

The MONA.health artificial intelligence system demonstrated 100% sensitivity and 99.81% specificity in detecting referable DR compared to the standard fundus camera and human DR grading. The positive predictive value (PPV) of 99.33% and positive likelihood ratio (LR+) of 528.00 reflect near-perfect concordance between MONA AI and the standard fundus camera in identifying patients with referable DR. Conversely, a negative predictive value (NPV) of 100% and a negative likelihood ratio (LR−) of 0.00 indicate complete agreement between MONA AI and the standard fundus camera in identifying patients without DR. The kappa (ĸ) value of 0.99 and diagnostic odds ratio (DOR) of infinity further support almost perfect agreement between MONA AI and the standard fundus camera with human grading in detecting referable DR, underscoring the exceptional test performance of MONA.health artificial intelligence. Finally, the diagnostic effectiveness (DE) of 99.85% compared to the standard camera demonstrates almost perfect accuracy of the MONA AI in correctly classifying subjects as having or not having referable DR (TP + TN) across all subjects.

### 3.3. Diagnostic Accuracy of the MONA.health Artificial Intelligence in the Detection of DME

**Figure 4:**
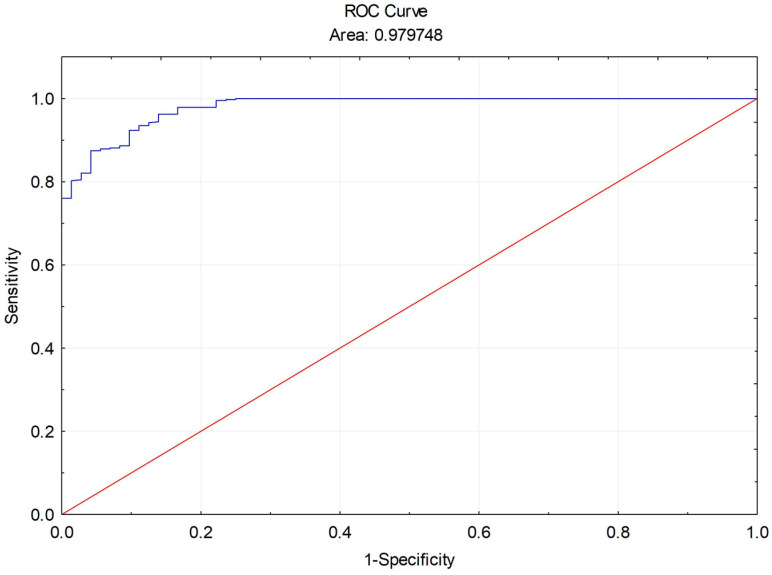
ROC curve of DME detection by the MONA AI vs. standard VISUCAM NM/FA Zeiss fundus camera.

Similar to the detection of referable DR, the shape of the ROC curve and the AUC of 97.97% demonstrate that the MONA.health artificial intelligence exhibits excellent discriminative power in identifying diabetic maculopathy compared with the standard VISUCAM NM/FA Zeiss fundus camera and human grading (Figure 4).

Additional diagnostic accuracy metrics for DME detection using the MONA AI, in comparison with the standard VISUCAM NM/FA Zeiss fundus camera and human grading, are presented in Table 3.

The MONA.health artificial intelligence system demonstrated 100% sensitivity and 98.95% specificity in detecting DME compared to the standard fundus camera and human grading. The positive predictive value (PPV) of 85.93% and positive likelihood ratio (LR+) of 95.67 reflect near-perfect agreement between MONA AI and the standard fundus camera in identifying patients with DME. Conversely, a negative predictive value (NPV) of 100% and a negative likelihood ratio (LR−) of 0.00 indicate complete concordance in identifying patients without DME. The kappa (ĸ) value of 0.81 and diagnostic odds ratio (DOR) of infinity further support substantial agreement among MONA AI, the standard fundus camera, and human grading in DME detection, underscoring the robust performance of the MONA.health artificial intelligence. Additionally, the diagnostic effectiveness (DE) of 99.02% compared to the standard camera demonstrates the high accuracy of MONA AI in classifying subjects as having or not having diabetic maculopathy (TP+TN) across all cases.

### 3.4. Real-Life Advantages of the MONA.health Artificial Intelligence

The MONA DR DME AI screening process demonstrated greater user-friendliness, requiring only 1 day of training and a median of 5 min for image capture and analysis. In contrast, the standard method required 7 days of training and a median of 13 min for image acquisition and grading (Table 4).

And finally, also essential and unavoidable, all fundus photographs, regardless of the camera used, were obtained by the ophthalmologically trained and experienced nurse Ružica Lucić. This approach allowed retina specialists additional time to perform other detailed diagnostic retinological procedures and to provide timely treatment for patients with advanced stages of diabetic retinopathy and macular edema, conditions that often require urgent intervention.

## 4. Discussion

This real-life study evaluates the performance of the deep learning model underlying the MONA.health software on fundus photography obtained with an automated, portable retinal camera, compared with standard fundus photography graded by retina specialists. The MONA.health artificial intelligence system demonstrated 100% sensitivity and 99.81% specificity in detecting referable DR, and 100% sensitivity and 98.95% specificity in detecting DME, compared with the standard fundus camera and human grading for DR and DME. These results surpass the predefined superiority thresholds of 85% sensitivity and 82.5% specificity previously proposed by Abràmoff et al. [16]. In their pivotal trial conducted in primary care offices at 10 sites, enrolling 900 participants of different ethnicities, the AI system achieved a sensitivity of 87.2% (95% CI, 81.8–91.2%) (>85%), specificity of 90.7% (95% CI, 88.3–92.7%) (>82.5%), and imageability rate of 96.1% (95% CI, 94.6–97.3%), demonstrating the capability of AI to provide specialized diagnostics in primary care environments. Based on these findings, the FDA authorized the system for use by health care providers to detect more than mild DR and DME, establishing it as the first FDA-authorized autonomous AI diagnostic system in any field of medicine and potentially helping to prevent vision loss in thousands of people with diabetes each year [11,16]. The present results are also consistent with a retrospective, systematic assessment of the MONA.health diabetic eye screening software, which uses artificial intelligence to analyze fundus images and integrates DR and DME classification outcomes into a single diagnostic output [20]. Furthermore, the present outcomes align with earlier published findings [21,22,23,24,25]. Such analyses are essential for evaluating the software’s real-world applicability in clinical settings and for identifying potential challenges in implementing AI-based clinical decision support systems.

The MONA.health software is officially approved in Europe for diabetic eye screening. To enable a more detailed evaluation of the software’s performance, analyses were stratified by ethnicity [20]. The most notable deviation was observed in sensitivity within the Caucasian subgroup, which reached 84.03%. It has been hypothesized that detecting DR may be more challenging in the Caucasian population due to a lower prevalence of certain retinal features (such as severe retinopathy or specific types of maculopathy) compared to other ethnic groups, which can make early-stage, subtle lesions harder to detect, and because dust artifacts and image shadows were sometimes misidentified as hemorrhages [26]. In the present study, there was no ethnic diversity, as all participants were Caucasian. The results indicate that the MONA.health artificial intelligence system achieved 100% sensitivity and 99.81% specificity in detecting referable DR compared to the standard fundus camera and human DR grading. Generally, sensitivity values range from 83.7% to 98.7% for referable DR and up to 99.8% for moderate-to-severe nonproliferative DR and PDR, which are similar to those reported by MONA.health and other automated retinal image analysis systems for DR detection [17]. Earlier standards, primarily based on expert consensus, suggested that detection rates for any form of DR with mobile retinal screening should not be lower than 90%, which is higher than the current threshold for human graders evaluating referable DR (85%) [27].

Agreement among human evaluators varies considerably, particularly when assessing early stages of DR and DME. Human evaluators miss 11% of cases with mild nonproliferative DR and 2–4% of cases with referable DR [28]. This variability may account for the differences in sensitivity observed in automated retinal image analysis systems, both between and within vendors, when evaluating early stages of DR and DME. In clinical settings, these variations are not considered significant, as the primary objective of screening is to identify patients who require immediate treatment. Among patients with referable DR confirmed by the reference standard and preserved visual acuity, automated retinal image analysis systems failed to identify fewer than 0.5% of cases that were later referred for DR or other ocular conditions. Consistent with previous reports, automated retinal image analysis systems such as MONA.health can serve as initial triage tools to classify patients into low-risk groups that do not require human grading and into medium- or high-risk groups that need further expert observation [12,29,30]. From this perspective, reduced specificity, corresponding to a higher false-positive rate, may remain economically justified, as it can expand grading capacity, even when a visual acuity criterion is applied. The use of automated retinal image analysis systems for screening approximately 2 million individuals each year could result in annual cost savings of up to €10 million, even with earlier versions that have lower test performance [30].

Many existing evaluations of automated retinal image analysis systems are limited by reliance on single-system assessments conducted in populations with limited sociodemographic variability, relatively small cohort sizes, and insufficient clarity regarding image pre-selection or preprocessing. Additionally, key methodological details such as image acquisition devices, grading procedures, and reference standards are often inadequately described [31]. However, a study that conducted direct head-to-head evaluations of multiple automated retinal image analysis systems using a large, well-powered, sociodemographically diverse, and clinically relevant dataset demonstrated high sensitivity for both medium- and high-risk DR within a real-world screening setting, with consistent performance across population subgroups [17].

In routine clinical practice, the implementation of multiple automated retinal image analysis systems across diverse populations is anticipated to achieve sensitivity comparable to or greater than that of human graders for moderate-to-severe nonproliferative and PDR, as well as for the detection of DME. These systems can be safely employed as triage tools in large-scale screening programs, potentially reducing the need for human grading by up to 80% [32] and decreasing the interval between diagnosis of vision-threatening DR and initiation of appropriate treatment. The effectiveness of artificial intelligence in the early detection of referable DR and DME, combined with the involvement of nurses and technicians in the screening process, will become increasingly important as the prevalence of diabetes is projected to rise significantly [2]. Nevertheless, several limitations must be acknowledged. The performance of AI systems depends on image quality and the characteristics of the training population, which may affect generalizability. Furthermore, AI algorithms serve as decision-support tools and should not supplant clinical judgment. Integration into existing clinical workflows, data protection, regulatory approval, and medico-legal responsibility also present significant practical challenges. Therefore, although MONA.health demonstrates considerable potential, its implementation should be accompanied by rigorous clinical oversight and validation in real-world settings.

The contributions of nurses and technicians to DR screening have been extensively documented in scientific literature, emphasizing their role in reducing the incidence of diabetes-related blindness and associated healthcare costs [33,34]. During DR screening, nurses and technicians not only optimize ophthalmologists’ time by enabling them to focus on patients who require treatment, but also provide ongoing education to patients about the importance of regular DR screenings and the management of risk factors such as glycemia, hypertension, and dyslipidemia to prevent vision loss. Providing nurses with a rapid, straightforward, and user-friendly device would enhance job satisfaction, increase motivation for patient education, and streamline daily tasks, thereby reducing the time required to obtain screening results. The present study identified a significant practical advantage: the MONA DR DME AI screening process was user-friendly, requiring only one day of training and a median of five minutes for image capture and analysis. In comparison, the standard method required seven days of training and a median of thirteen minutes for image acquisition and grading. Roux et al. recently reported a mean AI screening time of 11.7 min per patient, primarily for image acquisition, indicating that additional training could further reduce image-acquisition time even among skilled technicians [35].

Although the present results primarily reflect the technical performance of the MONA.health software, a comprehensive clinical evaluation is necessary to determine its real-world applicability and impact. In addition to accuracy metrics such as sensitivity and specificity, clinical validation should include assessment of workflow integration, user interaction, diagnostic concordance with ophthalmologists, patient outcomes, and cost-effectiveness. Prospective studies in diverse clinical settings are required to evaluate generalizability and to determine whether AI-assisted screening facilitates earlier detection, improves referral accuracy, and optimizes resource utilization. Thus, while the current findings support the software’s technical robustness, further real-world clinical studies are needed to confirm its clinical utility and long-term benefits.

Several limitations of the present study warrant consideration. First, the study was cross-sectional, conducted at a single health center, and included a small patient cohort. Second, the lack of ethnic diversity, with all participants being Caucasian, limits generalizability; external validation in more diverse populations is necessary given known variability in fundus pigmentation and image characteristics. Third, the absence of validation across multiple imaging modalities or data sources restricts assessment of the method’s generalizability and the algorithm’s robustness to noisy inputs or adversarial perturbations. Fourth, the relatively low prevalence of referable DR (13.7%) and DME (5%) in the cohort may affect predictive values and limit the robustness of certain estimates, particularly diagnostic odds ratios reported as “Infinity” due to zero false negatives. Additional methodological limitations include the absence of sample size justification and power calculation, lack of local cutoff validation, and a weak reference standard without assessment of inter-grader disagreement. Consequently, the high diagnostic performance observed should be interpreted with caution. The single-center design and limited sample size may influence performance estimates, and spectrum bias cannot be ruled out, especially if the distribution of disease severity differs from that in broader screening populations. Therefore, while these findings support the software’s robustness under the studied conditions, larger multicenter studies with more heterogeneous populations are required to confirm its performance and clinical utility.

## 5. Conclusions

This real-life clinical study demonstrated that the deep learning model underlying the MONA.health artificial intelligence software achieved high sensitivity and specificity for detecting referable DR and DME, comparable to those of standard fundus cameras and human grading. In routine clinical practice, deploying multiple automated retinal image analysis systems across diverse populations is anticipated to achieve sensitivity comparable to or greater than that of human graders for moderate-to-severe nonproliferative and PDR. These systems can be safely utilized as triage tools in large-scale screening programs, potentially reducing the need for human grading by up to 80%. Future implementation of artificial intelligence in DR screening will require clear, comprehensive legal and regulatory frameworks, particularly given the increasing global burden and prevalence of diabetes. It is crucial to foster trust among patients and healthcare professionals regarding the adoption of AI-based tools in DR screening. Transparency, accountability, and appropriate oversight will be essential to support the broad acceptance and safe integration of artificial intelligence into routine ophthalmological clinical practice.

## Figures and Tables

**Table 1:** Demographic data of patients included in the study.

	Total (*n* = 296)	T1DM (*n* = 56)	T2DM (*n* = 240)	Z	*p*-Value
Age (yr)	63 (19–81)	39 (19–60)	66 (31–81)	−10.708	**<0.001**
Gender (m/f) (%)	54/46	53.6/46.4	54.2/45.8	0.006	0.936
Diabetes duration (yr)	9 (1–39)	14.5 (1–34)	8 (1–39)	3.013	**0.003**

Legend: Values are medians (min-max) and percentages. Z and *p*-Values for comparison between patients with different types of diabetes. T1DM indicates type 1 diabetes mellitus; T2DM type 2 diabetes mellitus; yr: years.

**Table 2:** Diagnostic accuracy measures of referable DR detection by the MONA AI vs. standard VISUCAM NM/FA Zeiss fundus camera.

MONA DR vs.	VISUCAM NM/FA Zeiss Fundus Camera	
	Estimate	95% CI
Sensitivity	100%	94.40–100%
Specificity	99.81%	98.95–100%
PPV	99.33%	95.46–99.91%
NPV	100%	99.30–100%
LR+	528.00	74.51–3741.49
LR−	0.00	
Kappa ± SE	0.99 ± 0.01	0.97–1.00
DOR	Infinity	NaN-Infinity
DE	99.85%	99.10–100%

Legend: PPV indicates positive predictive value; NPV, negative predictive value; LR+, positive likelihood ratio; LR−, negative likelihood ratio; Kappa, kappa agreement; DOR, diagnostic odds ratio; DE, diagnostic effectiveness (accuracy); NaN—the calculation cannot be performed because the values entered include one or more instances of zero.

**Table 3:** Diagnostic accuracy measures of DME detection by the MONA AI vs. standard VISUCAM NM/FA Zeiss fundus camera.

MONA DME vs.	VISUCAM NM/FA Zeiss Fundus Camera	
	Estimate	95% CI
Sensitivity	100%	75.29–100%
Specificity	98.95%	97.74–99.62%
PPV	85.93%	73.37–93.12%
NPV	100%	99.35–100%
LR+	95.67	43.16–212.05
LR−	0.00	
Kappa ± SE	0.81 ± 0.08	0.66–0.96
DOR	Infinity	NaN-Infinity
DE	99.02%	97.84–99.65%

Legend: PPV indicates positive predictive value; NPV, negative predictive value; LR+, positive likelihood ratio; LR−, negative likelihood ratio; Kappa, kappa agreement; DOR, diagnostic odds ratio; DE, diagnostic effectiveness (accuracy); NaN—the calculation cannot be performed because the values entered include one or more instances of zero.

**Table 4:** Time required for image capture and analysis.

	MONA AI Screening Process	Standard Camera and Human Grading	Z	*p*-Value
Required time (min)	5 (3–8)	13 (10–19)	18.489	**<0.001**

Legend: Values are medians (min-max) and percentages. Z and *p*-Values for comparison between the time required for image capture and analysis with different devices and methods.

## Data Availability

The original contributions presented in this study are included in the article. Further inquiries can be directed to the corresponding author.
